# Cost-Effectiveness of 3D-Printed Patient-Specific Versus Off-the-Shelf Interbody Cages in Lumbar Spinal Fusion: A Markov Model Cost-Utility Analysis

**DOI:** 10.3390/jmahp14020018

**Published:** 2026-03-25

**Authors:** Jackson C. Hill, Ralph J. Mobbs, Marc Coughlan, Kevin A. Seex, Chloe A. Amaro, William R. Walsh, William C. H. Parr

**Affiliations:** 1 3DMorphic Pty Ltd., Matraville, NSW 2036, Australia; jhill@3dmorphic.com (J.C.H.); camaro@3dmorphic.com (C.A.A.); 2 Neuro Spine Clinic, Prince of Wales Private Hospital, Randwick, NSW 2031, Australia; 3 NeuroSpine Surgery Research Group (NSURG), Randwick, NSW 2031, Australia; 4 Coastal Neurosurgery, Gosford Private Hospital, Gosford, NSW 2250, Australia; 5 Macquarie Neurosurgery, Macquarie University Hospital, Macquarie Park, Sydney, NSW 2109, Australia; 6 Surgical and Orthopaedic Research Laboratories (SORL), Prince of Wales Clinical School, University of New South Wales (UNSW) Faculty of Medicine, Randwick, NSW 2031, Australia

**Keywords:** spinal fusion, cost-utility analysis, cost-effectiveness, 3D printing, Patient Specific Implant, Lumbar Interbody Fusion, reoperation rate

## Abstract

The aim of the present study was to compare the cost-effectiveness of 3DMorphic’s spinal 3DFusion Lumbar (3DFL) cages versus Off-The-Shelf (OTS) cages for patients undergoing lumbar interbody fusion in an Australian healthcare setting. 3DFL cages differ from generic OTS cages in that they are Patient-Specific Interbody Cages (PSICs). While several studies have discussed the clinical benefits of PSIC versus OTS cages, no studies have evaluated the cost-effectiveness of this technology. Without a direct randomised controlled trial between the two implant categories, an indirect treatment comparison was performed. The indirect comparison was informed by a clinical trial of 3DFL cages, the Australian Spine Registry and an analysis of reoperation rates for patients undergoing spinal fusion in an Australian cohort. In conclusion, the PSICs were demonstrated to be clinically superior to OTS cages as measured by Health Related Quality of Life (HRQoL) and reoperation rates. The cost-utility analysis demonstrated that 3DFL cages were cost-effective compared to OTS cages in an Australian healthcare setting.

## 1. Introduction

Cost-utility analysis (CUA) is a type of economic evaluation used to inform payors (e.g., governments, health funds, hospitals) when making reimbursement decisions on emerging technologies [1].

The Quality Adjusted Life Year (QALY) is used in CUA to quantify health outcomes [1]. The EQ-5D-5L questionnaire is a mechanism that derives utility values from patient reported health states by applying population-specific value sets [1,2,3,4].

By assessing differences in costs and health outcomes between treatment options, the Incremental Cost-Effectiveness Ratio (ICER) can be calculated. This ratio, expressed as cost per QALY gained, enables decision makers to assess whether an intervention represents value for money relative to their Willingness to Pay (WTP) thresholds. WTP thresholds represent the amount a healthcare system is willing to pay for 1 extra unit of health outcome (e.g., 1 total QALY) [1].

Lumbar Interbody Fusion (LIF) is a surgical treatment option used to address spinal pathologies including degeneration, trauma, instability and spinal deformities such as abnormal curvatures or alignment of the spine [5]. 3DFusion Lumbar (3DFL) cages (3DMorphic Pty Ltd., Sydney, Australia) have seen increasing clinical adoption in LIF procedures [6,7,8,9,10]. 3DFL cages differ to generic, Off-The-Shelf (OTS) cages as they are Patient-Specific Interbody Cages (PSICs). PSICs are made to match the anatomical contours of the patient’s vertebral endplates and provide the desired interbody alignment correction [6]. While several studies have discussed the clinical benefits of PSIC versus OTS cages, no studies have evaluated the cost-effectiveness of this new technology [6,7,8,9,11].

Reoperation rates for spinal fusion procedures are reportedly high, with one study reporting up to 18% in an Australian cohort within a two-year period [12]. Other recent studies, including a prospective clinical trial by Seex et al. of 78 patients, a 115 patient case series by Smith et al., and a 67 patient case series by Kent et al., demonstrated comparably low two-year reoperation rates for patients treated with PSIC of 3.8%, 4.3% and 4.5%, respectively [6,8,11].

The 2025 Seex et al. clinical trial also investigated Health Related Quality of Life (HRQoL) using the EQ-5D-5L questionnaire [6]. After an indirect comparison with the independently assessed Australian Spine Registry (ASR), the study concluded that there was an associated clinical benefit for patients treated with 3DFL cages over generic OTS cages [6].

Randomised Controlled Trials (RCT) for spinal fusion devices are limited and were not available for either PSIC or OTS cages in Australia. The use of strict inclusion and exclusion criteria, as well as the selection of a single OTS cage comparator, would limit generalisability of these studies to the broader patient population [13,14]. A conventional RCT of PSIC would also not be practical given the lack of randomisation and blinding with patient-specific manufacturing processes [13]. In the absence of RCT data, the aim of the present study was to use the best available evidence to assess whether 3DFL cages provide a cost-effective use of healthcare resources within the Australian healthcare system.

## 2. Materials and Methods

### 2.1. Study Overview

The present study reports on an indirect comparison of treatment for patients who received 3DFL cages and OTS cages in LIF. This cost-utility analysis was conducted considering the way in which Health Technology Assessments (HTA) are performed in Australia.

Ethics approval for the 2025 Seex et al. clinical trial of 3DFL cages was obtained from the South Eastern Sydney Local Health District (project identifier 2021/ETH11998) and the trial was registered with the Australian Therapeutic Goods Administration (CT-2022-CTN-00042-1) [6]. Informed consent was obtained from all the subjects involved in this study. The study was designed as a single region (Australian), multi-centre, multi-surgeon quantitative prospective cohort study of adult (>18 years) patients undergoing LIF with 3D printed titanium PSIC. Patients were followed up at 6-month, 12-month and 24-month intervals post-operatively.

Cost-effectiveness of 3DFL cages versus OTS cages was assessed over a two-year period for Australian patients requiring LIF for Degenerative Disc Disease (DDD). The perspective on clinical outcomes was from the patients receiving the device. The perspective on costs and economic outcomes was from the payers in the Australian healthcare system (private Health Insurers, state governments, and hospitals). The analysis took place over a two-year period, with no extrapolation of the data. To reflect the present value of costs and benefits incurred or received in the future, a uniform annual discount rate of 5% per year for all costs and health outcomes was used.

As part of the primary comparison, patient health outcomes (EQ-5D scores) were informed by the prospective clinical trial of 3DFL cages (Seex et al. 2025) and from the 1–2 level ALIF cohort of the 2024 Australian Spine Registry annual report [6,15]. The patient demographics are presented in Table 1.

The ICER was calculated to assess the required cost per additional unit health outcome (QALY) associated with 3DFL cages compared with the current standard of care [1]. The ICER was defined as the incremental cost divided by the incremental QALYs gained (see Equation (1)) [1]. Cost-effectiveness was assessed using an Australian WTP threshold of AUD50,000 per QALY gained [16]. An ICER below the AUD50,000/QALY threshold was considered cost-effective [1,17,18].

(1) $$ICER=\frac{Cost\;of\;new\;treatment-cost\;of\;comparator}{\left(QALY\;of\;new\;treatment-QALY\;of\;comparator\right)}$$

Equation (1): The equation to determine the ICER.

### 2.2. Model Development

#### 2.2.1. Literature Review

A literature review of existing studies reporting on the cost-effectiveness of spinal surgery was performed. No identified studies included an analysis of PSIC. Model structures within Raad et al. 2023 and Tirawanish et al. 2024 were used to inform the structure of the present model [19,20]. Utilities (QALYs), reoperation rates and costs were identified as clinically important and cost-driving factors [19,20]. A summary of the studies identified in the literature review are provided in Table 2, and a more detailed summary can be found in the Supplementary Materials Table S1.

#### 2.2.2. Model Structure

To evaluate the cost-effectiveness of 3DFL cages relative to OTS cages, we developed a state-transition (Markov) model using a one-year cycle length and a two-year time horizon [28,29]. The model structure was informed by the existing literature presented in Table 2 [19,20] and tailored to capture the clinically meaningful and cost-driving pathways relevant to Australian HTA requirements.

The model included four mutually exclusive health states:

1. No reoperation year 1: patients who have undergone surgery and have not required a reoperation within the first year. This utility value represents the HRQoL measured at the 12-month post-operative assessment.
2. No reoperation year 2: patients who remain reoperation free into the second year. This utility value represents the HRQoL measured at the 24-month post-operative assessment.
3. Reoperation: a non-absorbing state where patients may transition back to the no reoperation year 1 state, remain in reoperation, or transition to death.
4. Death: an absorbing state that can be reached from any other health state. Mortality transition probabilities were calculated based on the Australian Bureau of Statistics (ABS) mortality rates [30]. Mortality rates were uniformly applied to both the 3DFL and the OTS cohorts.

Due to constraints on the cycle length and time horizon, patients cannot transition past no reoperation year 2. Two-year reoperation rates were used to calculate annual transition probabilities, using the formula below [28]:

$$R=1-{\left(1-p\right)}^{\frac{1}{t}},$$

where *t* is the time period and *p* is cumulative probability over the respective period.

**Figure 1:**
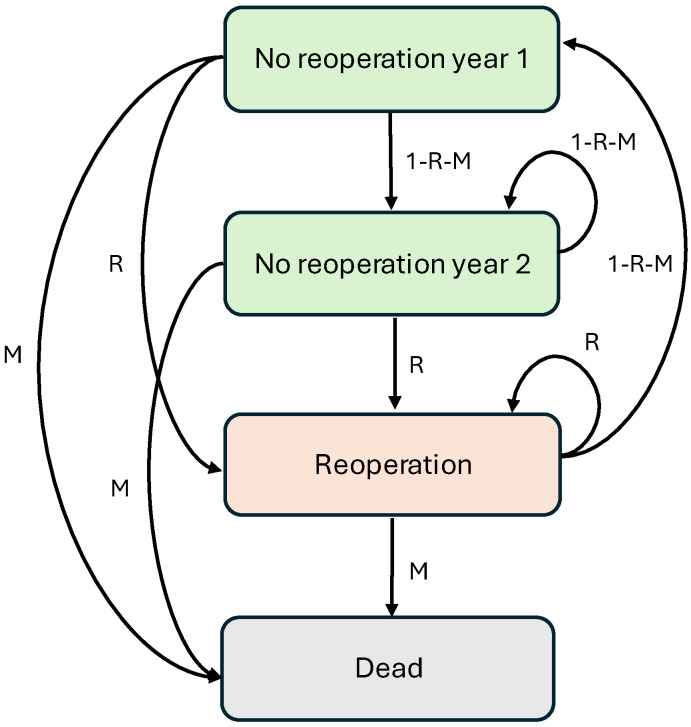
The Markov model structure used in the present economic evaluation. NB: M is the annual mortality rate, and R is the annual reoperation transition probability.

A diagrammatic illustration of the model used for this economic evaluation is provided in Figure 1.

#### 2.2.3. Structural Assumptions

All patients enter the model in the ‘no reoperation year 1’ health state immediately following initial surgery, meaning that no pre-operative health states were modelled. Patients can only move to ‘no reoperation year 2’ from the ‘no reoperation year 1’ health state. For simplicity and because modelling was performed over a two-year time horizon, if a patient requires a reoperation, their future reoperation risk is assumed to be the same as a patient who has not previously had a reoperation. The model structure and allowable transitions are the same for all comparator arms. Transition probabilities describe the probability of a patient transitioning from one state (e.g., no reoperation) to another state (e.g., reoperation, dead). Only the parameter values (utilities, transition probabilities and costs) differ between patient cohorts.

#### 2.2.4. Computational Methods

The Markov model was implemented using the Python programming language with Numpy, Pandas and matplotlib libraries (Python 3.11.3).

### 2.3. Model Parameters

#### 2.3.1. Utilities

Utility was measured by QALY values. The 1–2 level ALIF cohort from the 2024 ASR annual report was used to inform the utility values of the OTS cage comparator cohort [15]. These utility values are representative of a real-world cohort of patients treated with OTS LIF cages in the Australian healthcare setting. HRQoL values were derived from the EQ-5D-3L questionnaire by applying a value set described by Viney et al. 2011 [2]. This cohort is termed the ‘OTS ASR’ cohort and is used in the primary comparison between 3DFL cages and OTS cages.

For the 3DFL cages, utilities were informed by the Seex et al. 2025 prospective clinical trial [6]. HRQoL values were derived from the EQ-5D-5L questionnaire by applying a value set described by Norman et al. 2023 [3]. To control for any potential confounding of different pre-operative HRQoL states between the 3DFL cohort and the OTS ASR cohort, the 3DFL cohort was subsampled to a cohort of 40 patients whose preoperative HRQoL scores fell within ±0.3 of the ‘OTS ASR’ mean baseline. This subsampled cohort is termed the ‘3DFL ASR matched cohort’ and was used in the primary comparison between 3DFL cages and OTS cages.

A supplementary cohort of all patients from the Seex et al. 2025 study, regardless of pre-operative baseline utility scores, was also included [6]. This cohort is termed the ‘3DFL’ cohort.

To supplement the ‘OTS ASR’ cohort with another OTS device cohort, we used utility values from the literature search presented in Table 2 and Table 3. This cohort is termed the ‘OTS lit’ cohort.

The purpose of including the ‘3DFL’ and ‘OTS lit’ cohorts was to supplement the primary comparison and assess the consistency of the results by including these different scenarios.

#### 2.3.2. Costs

As costs were not reported in Seex et al. (2025), nor in the ASR 2024 annual report, cost data were derived from Lewin et al. (2021), a study conducted on a NSW cohort in the Australian healthcare system between 2010–2018 [6,12,15]. In Lewin et al. (2021), administrative data from the State Insurance Regulatory Agency was used to calculate costs per surgical episode and ongoing physical treatment costs up to 2 years post-op for lumbar spinal fusion [12]. The results were based on cost data from 3690 claims between 2010–2018, of which 1597 were lumbar fusion patients. Four cost categories were identified: direct surgical costs; medical treatment costs (pharmacy, anaesthesia, imaging); hospital costs (bedstay, operating theatre fees, surgical items, prosthesis, etc.); physical treatment costs (chiropractic, physiotherapy, etc.) [12]. These represent costs from an Australian health system payer perspective and do not include indirect costs such as lost work. Other potential cost savings associated with 3DFL cages such as reduced operating time and lower intraoperative complications were conservatively excluded [31,32].

Mean cost for a lumbar spinal fusion procedure was reported at AUD 52,379 (assumed as FY2017/18) [12]. Adjusted for inflation, the estimated mean cost of a spinal fusion procedure (with generic OTS cages) in FY2023/24 is AUD 63,867 [33]. In the present study, the only modelled difference in the cost of treatment between the 3DFL cohorts and the OTS cohorts was in the cost of the spinal fusion cage. Total operation cost with PSICs was modelled as AUD 75,763 (which includes a modelled premium for the new PSIC technology).

#### 2.3.3. Reoperation Rates

Reoperation rates were measured over a 2-year post-operative period and were defined to capture all surgical reoperations, irrespective of whether they were related to the interbody fusion cage. Reoperation rates for the 3DFL cohorts were derived from the Seex et al. (2025) clinical trial, which reported a reoperation rate of 3.8% [6].

The ASR 2024 annual report does not record reoperation rates [15]. As such, the reoperation rate identified in Lewin et al. (2021) was used for OTS cages (18%) [12]. In the absence of registry or RCT data on reoperation rates for spinal fusion patients in Australia, the Lewin et al. (2021) NSW cohort (2010–2018) provides the most relevant available [12].

#### 2.3.4. Model Parameters Summary

Table 4 provides a summary of all the parameters used in the CUA.

### 2.4. Model Validation

Traces were used to track patients through the model at each iteration to validate the model logic. For the ‘3DFL ASR matched’ patients, at the end of year 2, 95.3% of patients remained in the ‘no reoperation year 2’ (uncomplicated) health state. This is consistent with a 3.8% two-year reoperation rate, with a 0.9% two-year mortality rate.

### 2.5. Sensitivity Analyses

One-way, multivariate and probabilistic sensitivity analyses were performed to evaluate uncertainty in the model. In the one-way sensitivity analysis, 95% Confidence Intervals (CI) were used to vary the utility and cost parameters, which reflects the observed variability. For reoperation rates, a 25% variation in line with Vertuani et al. (2015) was assumed [34]. The 25% variability represents a conservative but published approach to defining upper and lower bounds as part of sensitivity analyses. The parameters and upper and lower bounds are presented in Table 5.

A Monte Carlo simulation was performed with 10,000 iterations as part of probabilistic sensitivity analyses. Probabilities and utilities were sampled from beta distributions, and costs were sampled from gamma distributions with 10% coefficient of variation [1,19].

## 3. Results

### 3.1. Incremental Cost-Effectiveness

The following results are from the perspective of the Australian healthcare system, with a time horizon of 2 years. The expected total QALYs gained per patient in the ‘3DFL ASR matched’ cohort were 1.65, and 1.3 for the ‘OTS ASR’ cohort. The expected total costs, as calculated from the Markov model, per patient were AUD 92,347 and AUD 88,857 for ‘3DFL ASR matched’ and ‘OTS ASR’, respectively. This means that the incremental difference in cost for 3DFL over 2 years was AUD 3490 (Table 6).

Total costs and QALYs for each group are presented in Table 6.

The ICER for the primary comparison (3DFL ASR matched vs. OTS ASR) was AUD 9971/QALY (Table 7). This means that for every extra QALY the 3DFL cages provide, there is an additional cost of AUD 9971 compared to operations using OTS devices, to the payer. The AUD 9971/QALY is well below the Australian WTP threshold of AUD 50,000/QALY [16].

### 3.2. Sensitivity Analyses

#### 3.2.1. One-Way Sensitivity Analysis

**Figure 2:**
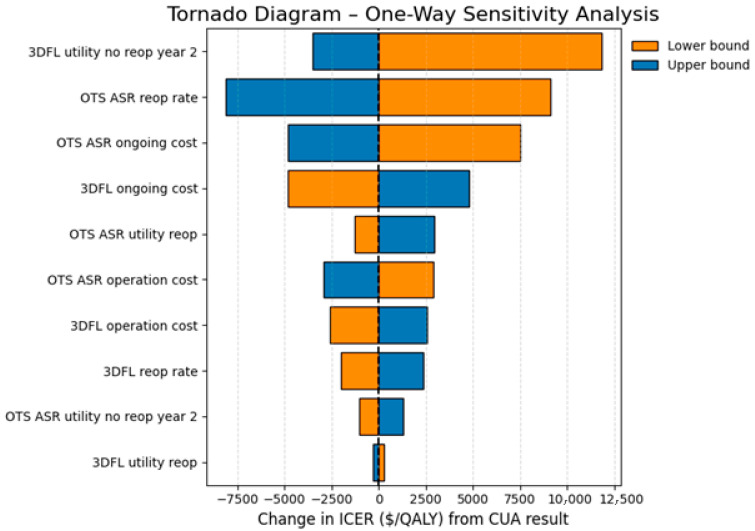
Tornado diagram demonstrating 3DFL ICER results from the one-way sensitivity analysis.

In the present study, one way sensitivity analysis identified; (1) the ‘3DFL ASR matched’ “utility no reoperation year 2” and (2) the OTS ASR “reoperation rate” parameters as the two most key drivers of the results. It is common for the results of cost-effectiveness analyses to be sensitive to small changes in utility values [1]. These results are presented in a Tornado diagram in Figure 2.

Reducing the utility values of the ‘3DFL ASR matched’ cohort to the lower end of the 95% CI increased the ICER to AUD 21,813/QALY.

Reducing the reoperation rate of the OTS ASR cohort to the lower bound (13.5% OTS reoperation rate) increased the ICER to AUD 19,076/QALY. Increasing the reoperation rate to the upper bound (22.5% OTS reoperation rate) reduced the ICER to AUD 1846/QALY).

The ICER remains below the Australian WTP threshold for all one-way sensitivity analysis checks, demonstrating that 3DFL cages remain cost-effective and below the recommended funding requirements (ICER < AUD 50,000/QALY) when tested against worst-case scenarios [16].

#### 3.2.2. Multivariate Sensitivity Analysis

Best and worst-case scenarios were simulated by simultaneously varying the utility values of the ‘3DFL ASR matched’ cohort and the reoperation rates of the ‘OTS ASR’ cohort to the upper and lower bounds (Table 5). In a worst-case scenario, combining pessimistic assumptions, the ICER rose to AUD 44,964/QALY. A best-case scenario combining optimistic scenarios reduced the ICER to AUD 1220/QALY.

#### 3.2.3. Probabilistic Sensitivity Analysis

A cost-effectiveness acceptability curve (CEAC) is used to demonstrate the probability that the 3DFL cage will remain cost-effective at different Willingness to Pay (WTP) thresholds [1,16,35]. In this analysis, the ‘3DFL ASR matched’ and OTS literature Cohorts were compared with the ‘OTS ASR’ cohort.

**Figure 3:**
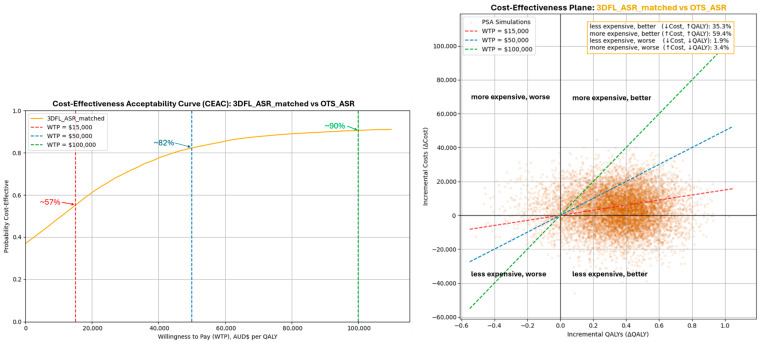
Cost-effectiveness acceptability curve (CEAC; **left**), and cost-effectiveness plane (**right**) for the ‘3DFL ASR matched’ cohort modelled against the ‘OTS ASR’ cohort. In the CEAC plot, at each intersection between the WTP (dashed) line and the CEAC curve, the probability that the 3DFL device is cost-effective is equivalent to the ratio of the number of points in the cost-effectiveness plane below the WTP line. In other words, for the WTP AUD 50,000 per QALY, approx. 0.82 (82%) of the simulated Probability Sensitivity Analysis (PSA) simulation points are under the blue dashed WTP line in the cost-effectiveness scatterplot (**right**).

WTP thresholds were selected to help visualise and interpret the results and are according to [1,16,17,18]. As the WTP values increase, there is an increasing probability that the 3DFL devices could be considered cost-effective. At the standard Australian WTP threshold of AUD 50,000/QALY, there is an approx. 0.82 probability (82% chance) the 3DFL device would be cost-effective compared to the ‘OTS ASR’ Cohort, as demonstrated by the CEAC curve in Figure 3 (left).

The cost-effectiveness plane in Figure 3 (right) demonstrates the probability that the subject device (3DFL cages) is cost-effective in providing better health outcomes (QALYs) compared to comparator OTS cages. The plane demonstrates that approximately 35% of the simulations fell in the bottom right (less expensive, better) quadrant, demonstrating that the intervention would cost less and provide higher QALYs. 60% of the simulations fell within the top right (more expensive, better) quadrant, demonstrating the intervention would cost more but provide higher QALYs.

## 4. Discussion

### 4.1. Clinical Outcomes

The reoperation rates and utility values used in the present study align closely with other literature reported results.

Reoperation rates were chosen to capture all surgical reoperations, irrespective of whether they were related to the interbody fusion cage. For the 3DFL cohort, reoperation rates were derived from Seex et al. (2025), a clinical trial that reported a 3.8% reoperation rate at two years follow-up [6]. Comparable rates for PSIC have been reported by Smith et al. (2025) at 4.3% (two years) and Kent et al. (2024) at 4.5% (15-month median follow-up) [8,11].

Published reoperation rates following the use of OTS cages vary considerably. Some US based studies, such as Guyer et al. (2023) and Hosseini et al. (2017) have reported relatively high reoperation rates of 15.5% and 28%, respectively [36,37]. Other cohorts, including those reported by Kashlan et al. (2020) and Siepe et al. (2015) demonstrate substantially lower rates of 4.9% and 5.6%, respectively [38,39]. The base case reoperation rate of 18% for the OTS ASR cohort in the present study was informed by Lewin et al. (2021), which provides the most relevant real-world Australian evidence and is consistent with US reported reoperation rates (15.5–28%) [12,36,37]. As part of the sensitivity analysis, a reoperation rate of 13.5% for OTS cages was modelled. This resulted in an ICER value of AUD 19,076/QALY, which is still below the implicit Australian WTP value of AUD 50,000/QALY [16,35].

HRQoL scores that were calculated from the EQ-5D-5L questionnaire in Seex et al. (2025) were used as utility values for the 3DFL patients (Table 4) [6]. Comparator utility values were derived from the ASR [15].

An additional supplementary cohort of OTS patients was derived from the literature review presented in Table 3. The utility values for this supplementary cohort ranged from 0.57–0.87, with a mean of 0.69 at both 1 and 2 years post-op. Although these studies included in this cohort used the same surgical technique (circumferential fusion), the heterogeneity in utility values likely reflects differences in study location and the utility measurement instruments. The mean utility value of 0.69 at both 1 and 2 years post-op was consistent with the ASR values (0.70 at 1 year and 0.71 at 2 years post-op) [15].

### 4.2. Cost

Cost inputs for the present study were derived from Lewin et al. (2021) [12]. There was a lack of studies reporting on the cost-effectiveness of lumbar spinal fusion in an Australian setting, meaning that costs could not be sourced from the literature studies presented in Table 2. Despite this, the costs applied in the present CUA were consistent with values reported internationally. Virk et al. (2012) reported costs between USD 37,646 to USD 48,742 [26]. By applying 2012 exchange rates and adjusting for inflation, these costs are estimated to be between AUD 48,751 and AUD 63,120 [33]. The costs for a patient receiving an OTS cage in the present study was AUD 63,867 [12,33]. For simplicity, the only modelled difference in operation cost between 3DFL and OTS cohorts was in the price of the cage. Other potential cost savings associated with 3DFL, such as reduced operating time or lower intraoperative complications were not included [32,40]. This means that the models presented here represent conservative scenarios and may underestimate the cost advantages of 3DFL relative to OTS cages.

### 4.3. Cost-Effectiveness

The ICER of the base case was AUD 9971/QALY. This reflects the incremental difference is prothesis costs between the two comparators, weighted against the difference in clinical outcomes as measured by QALYs and reoperation rates. A commonly cited WTP threshold reported in the literature for the Australian healthcare system is AUD 50,000/QALY [1,16,17,18,35]. Considering a AUD 50,000/QALY threshold for cost-effectiveness, the resulting ICER of AUD 9971/QALY for 3DFL cages demonstrates cost-effectiveness.

While the 3DFL cage was modelled to increase the estimated total cost of care per patient (AUD 92,347 vs. AUD 88,857), the additional costs are offset by the incremental QALY gains. The ICER of the primary comparison in the economic evaluation was AUD 9971/QALY, well below the commonly cited Australian WTP threshold of AUD 50,000/QALY [1,16,17,18,35]. The cost-effectiveness of 3DFL cages was driven by the superior patient outcomes (QALY) and reduced reoperation rates relative to OTS cages.

### 4.4. Sensitivity Analyses

Probabilistic sensitivity analysis showed that in over a third (approx. 35%) of the simulations, 3DFL cages were both more effective and less costly than comparator technologies. At a AUD 50,000/QALY threshold, the cost-effectiveness acceptability curve (CEAC) demonstrated an approx. 82% probability that 3DFL cages are cost-effective. This probability remained above 50% (approx. 57%) at a AUD 15,000/QALY threshold and approached 90% at the higher AUD 100,000/QALY WTP threshold.

One-way sensitivity analysis demonstrated that the model was most sensitive to the utility values of the 3DFL cohort. Even when this parameter was reduced to the lower bound of the 95% confidence interval, the ICER remained below the AUD 50,000/QALY threshold at AUD 21,813/QALY (Figure 2).

### 4.5. Limitations and Bias

In the absence of RCT data directly comparing 3DFL and OTS cages, we aimed to use the most relevant real-world datasets in this CUA. Inputs for the comparative effectiveness were derived from an indirect treatment comparison rather than from head-to-head trial data. Comparator health outcome data were informed by the ASR, which captures real-world outcomes across a broad mix of OTS fusion cage designs and clinical practice patterns. Confounding was controlled for by subsampling the 3DFL cohort as described in Section 2.3.1; however, residual differences in patient age, surgeon, hospital and other potential confounders remain. Although indirect comparisons are susceptible to confounding and cross-cohort differences that may not be fully adjusted for, head-to-head trial evidence between 3DFL cages and a single OTS cage design would have limited generalisability to the broader set of generic OTS cages use in practice.

The two-year model horizon may underestimate the total costs and total QALYs gained. The one-year cycle limits the level of granularity in the model. The one-year cycle was selected as clinical outcome data were available at one-year intervals. The four-state structure simplifies the clinical pathway and does not capture post-reoperation recovery states or differentiate between primary and secondary reoperations.

The cost-effectiveness analysis presented here was designed considering the way in which HTA are performed in Australia. Accordingly, all inputs (costs, utilities, WTP thresholds and reoperation rates) reflect the Australian setting. Generalisability to other healthcare settings may therefore be limited, which is inherent to cost-effectiveness analyses.

## 5. Conclusions

The results presented in this economic evaluation indicate that, at the modelled benefit, the 3DFL cage is a cost-effective alternative to generic OTS cages for Australian patients.

The findings here are specific to 3DFL cages.

## Figures and Tables

**Table 1:** Patient demographics, population characteristics and intervention characteristics from the Seex et al. 2025 clinical trial and the 1–2 ALIF cohort from the Australian Spine Registry [6,15].

Patient Demographics	3DFL [6]	OTS ASR [15]
Sample Size	78	146
% Male/Female	56%	60%
Age (median)	60.5	48
Population Characteristics	Adult (>18 years old) patients were selected for surgery due to discogenic and/or mechanical low back pain symptomatic of DDD. The mean number of operated levels was 1.4.	Adult (>18 years old) patients for this cohort were included based on surgical intervention (1–2 level ALIF) instead of pathology. The number of operated levels was less than or equal to 2.
Intervention Characteristics	Lumbar Interbody Fusion (LIF). Anterior (ALIF) and lateral (ATP/LLIF/OLIF/XLIF) approaches. The patients underwent anterior column LIF with some cases supplemented by posterior fixation (circumferential fusion). Posterior only approaches (e.g., PLIF or TLIF) were excluded.	Lumbar Interbody Fusion (LIF). 1–2 Level ALIF. Patients were excluded if they had a decompression, posterior surgery, or if the surgery was a staged operation.

**Table 2:** Summary of the literature review of cost-effectiveness studies in spinal fusion.

Author & Year	Type of Economic Evaluation	Key Findings
Raad et al. 2023 [19]	CUA	The model used health states of ‘uncomplicated’, ‘revision’ or ‘dysphagia’. A USD 50,000/QALY gained societal Willingness To Pay (WTP) threshold was assumed for the analysis.
Tirawanish et al. 2024 [20]	CUA	The model structure included health states of ‘well’, ‘complicated’ and ‘death’. Utilities for a 12-month post-operative successful outcome were 0.854 and 0.882 for LLIF and PLIF patients, respectively. Utilities for patients requiring a revision were 0.671 and 0.646 for LLIF and PLIF patients, respectively.
Soegaard et al. 2007 [21]	CUA	The utility values at long term follow up were 0.48 and 0.59 for the posterolateral and circumferential group, respectively. The reoperation rate for the circumferential cohort was 15%.
Freeman et al. 2007 [22]	Cost-Effectiveness Analysis (CEA)	Mean utility levels were 0.57 at 1 and 2 years post-op for the titanium cage. 17.1% of patients with the titanium cage required revision surgery.
Jazini et al. 2018 [23]	CEA	The cost of index surgery for the circumferential group was USD 31,466. 12.9% readmission rate for the circumferential cohort.
Tosteson et al. 2008 [24]	CEA	2-year total QALYs gained for circumferential fusion patients was 1.62.
Passias et al. 2021 [25]	Cost–Benefit Analysis	12-month EQ-5D scores were 0.60, 0.81 and 0.63 for open, MIS and robot surgery patients, respectively. Procedure costs were USD 42,539, USD 41,171, and USD 60,047 for open, MIS and robot surgery patients, respectively. Revision rates were (3% open, 3% MIS and 5% robotic surgery) at 1-year post-op.
Virk et al. 2012 [26]	CEA	QALY values for patients experiencing a successful fusion surgery and a failed fusion surgery were 0.66 and 0.27, respectively. In the first two years, annual revision rates varied between 2–6.87%. Costs were reported between USD 37,646 to USD 48,726 (in 2012).
Khan et al. 2022 [27]	CEA	12-month EQ-5D scores were 0.733 for the Tritanium cages. 90-day readmission rates were 7.9% for the Tritanium cage. Average cost of surgery was calculated to be USD 29,194.90 (in 2021).

**Table 3:** Published cost-effectiveness literature used to derive utility values for the ‘OTS_Lit’ cohort.

Study Authors	Study Type	Cohort Characteristics	Intervention	Utility 1 Year Post Op	Utility 2 Years Post Op
Virk et al. 2012 [26]	Cost-effectiveness of graft options in spinal fusion surgery	Developed a Markov model to identify the most cost-effective graft option for use in spinal fusion to treat 1 level degenerative spondylolisthesis. The authors divide outcomes for both successful fusion and failed fusion.	Lumbar fusion	0.66	0.66
Tirawanish et al. 2024 [20]	Cost-effectiveness of LLIF compared with PLIF in lumbar fusion in Thailand	Patients were treated for lumbar spondylosis with back and leg pain. The operative technique involved LLIF with a PEEK cage at a single level between 2014–2020 performed by a single surgeon with supplementary posterior fixation.	Circumferential fusion	0.87	0.85
Freeman et al. 2007 [22]	Cost-effectiveness of two forms of circumferential lumbar fusion	Cost-effectiveness of a titanium cage vs. femoral ring allograft in lumbar spinal fusion.	Circumferential fusion	0.57	0.57
Jazini et al. 2018 [23]	Cost-effectiveness of circumferential fusion for lumbar spondylolisthesis	Cost-effectiveness of circumferential fusion vs. TLIF (circumferential cohort reported here).	Circumferential fusion	0.65	0.65
Soegaard et al. 2007 [21]	Cost-utility evaluation of circumferential fusion versus posterolateral fusion	A Randomised Controlled Trial setting for patients with severe chronic low back pain. The study compared circumferential fusion versus posterolateral fusion (circumferential cohort reported here).	Circumferential fusion	0.59	0.59
Tosteson et al. 2008 [24]	Cost-effectiveness	Cost-effectiveness of surgical treatment for patients with spinal stenosis with and without degenerative spondylolisthesis	Circumferential fusion	0.81	0.81
Average				0.69	0.69

**Table 4:** Summary of the model parameters used within each patient cohort. The cohorts used in the primary comparison are denoted by *.

Parameter Type	Patient Cohort	Model Parameter	Value	Reference	Additional Info/Assumptions
Utility	3DFL	No reoperation year 1	0.83	Seex et al. (2025) [6]	HRQoL score for patients who were ‘well’, not requiring a reoperation at one-year post-op.
		No reoperation year 2	0.82	Seex et al. (2025) [6]	HRQoL score for patients who were ‘well’, not requiring a reoperation at two years post-op.
		Reoperation	0.31	Seex et al. (2025) [6]	HRQoL score for patients who required reoperation.
	3DFL (ASR matched) *	No reoperation year 1	0.84	Seex et al. (2025) [6]	Subsampled cohort of 40 patients, stratified by preoperative baseline to within ± 0.3 of the mean ASR preoperative baseline HRQoL index.
		No reoperation year 2	0.86	Seex et al. (2025) [6]	
		Reoperation	0.31	Seex et al. (2025) [6]	HRQoL score for patients who required reoperation.
	ASR *	No reoperation year 1	0.70	Australian Spine Registry 2024 report [15]	These numbers are derived from the 1–2 level ALIF group in the ASR.
		No reoperation year 2	0.71	Australian Spine Registry 2024 report [15]	These numbers are derived from the 1–2 level ALIF group in the ASR.
		Reoperation	0.31	Seex et al. (2025) [6]	As utility values (HRQoL scores) for patients undergoing reoperation were unavailable in the literature, we assumed equivalent utility between the ASR and 3DFL cohorts for this health state.
	Literature cohort	No reoperation year 1	0.69	Virk et al. (2012), Tirawanish et al. (2024), Freeman et al. (2007), Jazini et al. (2018), Soegaard et al. (2007), Tosteson et al. (2008) [20,21,22,23,24,26]	Average across multiple cost-effectiveness studies on lumbar spinal fusion.
		No reoperation year 2	0.69	Virk et al. (2012), Tirawanish et al. (2024), Freeman et al. (2007), Jazini et al. (2018), Soegaard et al. (2007), Tosteson et al. (2008) [20,21,22,23,24,26]	Average across multiple cost-effectiveness studies on lumbar spinal fusion.
		Reoperation	0.27	Virk et al. (2012) [26]	Virk et al. report 0.27 utility for unwell patients following spinal fusion.
2-year reoperation rate	3DFL	R	3.8%	Seex et al. (2025) [6]	3DFL cage reoperation rate reported in Seex et al. (2025) [6].
	3DFL (ASR matched) *	R	3.8%	Seex et al. (2025) [6]	3DFL cage reoperation rate reported in Seex et al. (2025) [6].
	ASR *	R	18%	Lewin et al. (2021) [12]	Reoperation data of an Australian (NSW) patient cohort.
	Literature cohort	R	18%	Lewin et al. (2021) [12]	Reoperation data of an Australian (NSW) patient cohort.
Costs	3DFL	Cost 3DFL operation	AUD 75,763	Lewin et al. (2021) [12], RBA inflation calculator [33] + PSIC premium	Operation costs from a health system (payer) perspective.
	3DFL (matched to ASR) *	Cost 3DFL operation	AUD 75,763	Lewin et al. (2021) [12], RBA inflation calculator [33] + PSIC premium	Operation costs from a health system (payer) perspective.
	ASR *	Cost ASR operation	AUD 63,867	Lewin et al. (2021) [12], RBA inflation calculator [33]	Operation costs from a health system (payer) perspective.
	Literature Cohort	Cost ASR operation	AUD 63,867	Lewin et al. (2021) [12], RBA inflation calculator [33]	Operation costs from a health system (payer) perspective.
	All groups *	Ongoing medical costs up to two years	AUD 7106 per year	Lewin et al. (2021) [12], RBA inflation calculator [33]	Ongoing medical related costs from a health system (payer) perspective. These were assumed to be consistent across all cohorts.

**Table 5:** Parameters and their respective upper and lower bounds used in the sensitivity analysis.

Patient Cohort	Parameter	Value	Lower Bound (95% CI)	Upper Bound (95% CI)
3DFL ASR matched	Utility no reoperation year 1	0.84	0.778	0.912
	Utility no reoperation year 2	0.86	0.762	0.96
	Utility reoperation	0.31	0	0.754
	Reoperation rate	3.8%	2.85%	4.75%
	Operation cost	AUD 75,763	AUD 74,895	AUD 76,631
	Ongoing yearly costs	AUD 7106	AUD 6238	AUD 7974
OTS ASR	Utility no reoperation year 1	0.7	0.672	0.727
	Utility no reoperation year 2	0.71	0.682	0.737
	Utility reoperation	0.31	0	0.754
	Reoperation rate	18%	13.5%	22.5%
	Operation cost	AUD 63,867	AUD 62,999	AUD 64,735
	Ongoing yearly costs	AUD 7106	AUD 6238	AUD 7974

**Table 6:** Total costs and total QALYs gained at the 2-year post-op time point across the four different scenarios.

Subgroup	Total Costs	Total QALYs per Patient
3DFL	AUD 92,347	1.57
3DFL ASR matched	AUD 92,347	1.65
OTS ASR	AUD 88,857	1.3
OTS Literature	AUD 88,857	1.26

**Table 7:** Incremental Cose Effectiveness Ratio’s (ICERs) calculated between each modelled cohort.

Compared Technologies	ICER Value
3DFL ASR matched vs. OTS ASR ^1^	$ICER=\frac{\$92,347-\$88,857}{1.65-1.3}=AUD\;9971/{QALY}^{\;1}$
3DFL vs. OTS ASR	$ICER=\frac{\$92,347-\$88,857}{1.57-1.3}=AUD\;12,926/QALY$
3DFL vs. OTS Literature	$ICER=\frac{\$92,347-\$88,857}{1.57-1.26}=AUD\;11,258/QALY$
3DFL ASR matched vs. OTS Literature	$ICER=\frac{\$92,347-\$88,857}{1.65-1.26}=AUD\;8949/QALY$

^1^ The result from the primary comparison.

## Data Availability

The original contributions presented in this study are included in the article/Supplementary Materials. Further inquiries can be directed to the corresponding author.

## Supplementary Materials

The following supporting information can be downloaded at: https://www.mdpi.com/article/10.3390/jmahp14020018/s1. Table S1: Summary of the literature review of cost-effectiveness studies in spinal fusion.

## Abbreviations

The following abbreviations are used in this manuscript:

CUA	Cost-Utility Analysis
QALY	Quality Adjusted Life Year
ICER	Incremental Cost-Effectiveness Ratio
WTP	Willingness to Pay
LIF	Lumbar Interbody Fusion
3DFL	3DFusion Lumbar
OTS	Off-The-Shelf
PSIC	Patient Specific Interbody Cages
HRQoL	Health Related Quality of Life
ASR	Australian Spine Registry
DDD	Degenerative Disc Disease
ALIF	Anterior Lumbar Interbody Fusion
ATP	Anterior to Psoas
LLIF	Lateral Lumbar Interbody Fusion
OLIF	Oblique Lumbar Interbody Fusion
XLIF	Xtreme Lumbar Interbody Fusion
PLIF	Posterior Lumbar Interbody Fusion
TLIF	Transforaminal Lumbar Interbody Fusion
ACDF	Anterior Cervical Discectomy and Fusion
CEA	Cost-Effectiveness Analysis
PEEK	Polyetheretherketone
ABS	Australian Bureau of Statistics
CEAC	Cost-Effectiveness Acceptability Curve
RCT	Randomised Controlled Trial
PSA	Probability Sensitivity Analysis
HTA	Health Technology Assessment
