# Supplementary material for: Cost-Effectiveness of 3D-Printed Patient-Specific Versus Off-the-Shelf Interbody Cages in Lumbar Spinal Fusion: A Markov Model Cost-Utility Analysis
Source: J Mark Access Health Policy. 2026 Mar 25;14(2):18. doi: 10.3390/jmahp14020018 (PMC13108168; doi:10.3390/jmahp14020018)
Supplement: Supplementary file 1 [file jmahp-14-00018-s001.zip › jmahp-4167414-supplementary.pdf]

Table S1: Summary of the literature review of cost-effectiveness studies in spinal fusion.

Author & Year	Article Title	Type of Economic Evaluation	Summary of Findings (model structure, utilities, reoperation rates and costs)
Raad et al. 2023 [19]	A Five-Year Cost-Utility Analysis Comparing Synthetic Cage Versus Allograft Use in Anterior Cervical Discectomy and Fusion (ACDF) Surgery for Cervical Spondylotic Myelopathy	CUA	• Raad et al. constructed a decision-analytic model for a patient undergoing ACDF with the choice of receiving either allograft or a Synthetic Cage. The model used health states of 'uncomplicated', 'revision' or 'dysphagia'.<br>• For an uncomplicated ACDF, patients were assumed to gain a health utility of 0.073 over each 12-month period.<br>• A $50,000/QALY gained societal willingness to pay (WTP) threshold was assumed for the analysis.<br>• Average cost per QALY gained was USD74,110/QALY and USD83,400/QALY for the allograft and the synthetic cage, respectively.
Tirawanish et al. 2024 [20]	Cost-Effectiveness and clinical outcomes of lateral lumbar Interbody Fusion With Tricalcium Phosphate and Iliac Bone Graft Compared With Posterior Lumbar Interbody Fusion With Local Bone Graft in Single-Level Lumbar Spinal Fusion Surgery in Thailand	CUA	• The CUA utilised a Markov model with a lifetime horizon and a societal perspective. The model structure included health states of 'well', 'complicated' and 'death'.<br>• Utilities for patients with a successful outcome past 12 months post-op were 0.854 and 0.882 for LLIF and PLIF patients, respectively.<br>• Utilities for patients requiring a revision were 0.671 and 0.646 for LLIF and PLIF patients, respectively.
Soegaard et al. 2007 [21]	Circumferential Fusion Is Dominant Over Posterolateral Fusion in a Long-term Perspective	CUA	• This study investigated incremental cost per QALY between circumferential fusion and posterolateral fusion from a societal perspective.<br>• The utility values at long term follow up were 0.48 and 0.59 for the posterolateral and circumferential group, respectively.<br>• Reoperation rates were 38% and 15% for the posterolateral and circumferential groups, respectively.
Freeman et al. 2007 [22]	ISSLS Prize Winner: Cost-Effectiveness of Two Forms of Circumferential Lumbar Fusion: A Prospective Randomized Controlled Trial	Cost Effectiveness Analysis (CEA)	• Freeman et al. evaluated cost effectiveness of a titanium cage versus a femoral ring allograft used in circumferential fusion.<br>• Mean utility levels were 0.57 at 1 and 2 years post-op for the titanium cage.<br>• 17.1% of patients with the titanium cage required revision surgery, and 8.1% of the femoral ring allograft patients.

Jazini et al. 2018 [23]	Cost-effectiveness of circumferential fusion for lumbar spondylolisthesis: propensity-matched comparison of transforaminal lumbar interbody fusion with anterior-posterior fusion	CEA	• A cost effectiveness comparison between single level TLIF and single level circumferential fusion.<br>• The cost of the index surgery was USD31,466 and USD29,425 for the circumferential and TLIF groups, respectively.<br>• 12.9% readmission rate for both circumferential and TLIF groups.
Tosteson et al. 2008 [24]	Surgical Treatment of Spinal Stenosis with and without Degenerative Spondylolisthesis: Cost-Effectiveness after 2 Years	CEA	• This study assessed the short-term cost effectiveness of spine surgery relative to nonoperative care for spinal stenosis alone and spinal stenosis with spondylolisthesis.<br>• 2-year total QALYs gained for circumferential fusion patients was 1.62.
Passias et al. 2021 [25]	A cost benefit analysis of increasing surgical technology in lumbar spine fusion	Cost Benefit Analysis	• This study compared economic outcome of lumbar spine fusion between open, minimally invasive surgery (MIS) and robot assisted surgery.<br>• 12-month EQ-5D scores were 0.60, 0.81 and 0.63 for open, MIS and robot surgery patients, respectively.<br>• Procedure costs were USD42,539, USD41,171, and USD60,047 for open, MIS and robot surgery patients, respectively.<br>• Revision rates were (3% open, 3% MIS and 5% robotic surgery) at 1-year post-op.
Virk et al. 2012 [26]	Cost Effectiveness Analysis of Graft Options in Spinal Fusion Surgery Using a Markov Model	CEA	• QALY values for patients experiencing low back pain, a successful fusion surgery, a failed fusion surgery and a successful revision surgery were 0.36, 0.66, 0.27 and 0.42, respectively.<br>• In the first two years, annual revision rates varied between 2-6.87%. Reoperation was not reported.<br>• Costs were reported between USD37,646 to USD48,726 (in 2012).
Khan et al. 2022 [27]	Clinical and Cost-Effectiveness of Lumbar Interbody Fusion Using Tritanium Posterolateral Cage (vs. Propensity-Matched Cohort of PEEK Cage)	CEA	• 12-month EQ-5D scores were 0.733 and 0.723 for the Tritanium and control TLIF cages, respectively.<br>• Direct (hospital) and indirect (societal) costs were calculated.<br>• 90-day readmission rates were 7.9% for the Tritanium cage and 4.39% for the TLIF control.<br>• Average cost of surgery was calculated to be USD29,194.90 (in 2021).
